# Fatty acid binding protein deletion suppresses inflammatory pain through endocannabinoid/*N*-acylethanolamine-dependent mechanisms

**DOI:** 10.1186/s12990-015-0056-8

**Published:** 2015-08-28

**Authors:** Martin Kaczocha, Sherrye T. Glaser, Thomas Maher, Brendan Clavin, John Hamilton, Joseph O’Rourke, Mario Rebecchi, Michelino Puopolo, Yuji Owada, Panayotis K. Thanos

**Affiliations:** Department of Anesthesiology, Stony Brook University, Stony Brook, NY 117940 USA; Department of Biological Sciences, Kingsborough Community College, Brooklyn, NY 11235 USA; Department of Psychology, Stony Brook University, Stony Brook, NY 11794 USA; Department of Organ Anatomy, Tohoku University Graduate School of Medicine, Sendai, Japan

**Keywords:** Fatty acid binding protein, FABP, Endocannabinoid, Anandamide, Pain

## Abstract

**Background:**

Fatty acid binding proteins (FABPs) serve as intracellular carriers that deliver endocannabinoids and *N*-acylethanolamines to their catabolic enzymes. Inhibition of FABPs reduces endocannabinoid transport and catabolism in cells and FABP inhibitors produce antinociceptive and anti-inflammatory effects in mice. Potential analgesic effects in mice lacking FABPs, however, have not been tested.

**Findings:**

Mice lacking FABP5 and FABP7, which exhibit highest affinities for endocannabinoids, possessed elevated levels of the endocannabinoid anandamide and the related *N*-acylethanolamines palmitoylethanolamide and oleoylethanolamide. There were no compensatory changes in the expression of other FABPs or in endocannabinoid-related proteins in the brains of FABP5/7 knockout mice. These mice exhibited reduced nociception in the carrageenan, formalin, and acetic acid tests of inflammatory and visceral pain. The antinociceptive effects in FABP5/7 knockout mice were reversed by pretreatment with cannabinoid receptor 1, peroxisome proliferator-activated receptor alpha, and transient receptor potential vanilloid 1 receptor antagonists in a modality specific manner. Lastly, the knockout mice did not possess motor impairments.

**Conclusions:**

This study demonstrates that mice lacking FABPs possess elevated levels of *N*-acylethanolamines, consistent with the idea that FABPs regulate the endocannabinoid and *N*-acylethanolamine tone in vivo. The antinociceptive effects observed in the knockout mice support a role for FABPs in regulating nociception and suggest that these proteins should serve as targets for the development of future analgesics.

## Findings

### Background

Fatty acid binding proteins (FABPs) are intracellular lipid chaperones that are expressed in cells of the central and peripheral nervous system [1–3]. In addition to fatty acids, FABPs interact with other endogenous lipids including the endocannabinoids, anandamide (AEA) and 2-arachidonoylglycerol (2-AG), and related *N*-acylethanolamines (NAEs) [4–7]. Endocannabinoids activate cannabinoid receptor 1 (CB1) to produce antinociceptive effects [8, 9]. Similarly, the NAEs palmitoylethanolamide (PEA) and oleoylethanolamide (OEA) activate peroxisome proliferator-activated receptor alpha (PPARα) centrally and peripherally to produce analgesic and anti-inflammatory effects [10, 11].

We have previously shown that FABP5 and FABP7 bind to endocannabinoids/NAEs with high affinities [5, 12]. FABPs mediate the intracellular trafficking of endocannabinoids/NAEs to their catabolic enzyme(s) such as fatty acid amide hydrolase (FAAH) [4]. Consequently, inhibition of FABPs reduces endocannabinoid uptake and inactivation [4]. Recently, we have shown that pharmacological inhibition of FABPs elevates brain levels of AEA. Similarly, others have demonstrated that mice lacking FABP5 likewise possess elevated brain AEA levels [6, 13]. Pharmacological inhibition of FABPs produces antinociceptive effects that are mediated by both CB1 and PPARα, suggesting the involvement of endocannabinoids and NAEs such as PEA [6, 14].

To date, it is not known whether mice deficient in FABP5 and FABP7 exhibit alterations in NAE and endocannabinoid levels and/or show altered nociception. Herein, we characterize the endocannabinoid system in mice lacking both FABP5 and FABP7 and provide evidence that these FABPs regulate the endocannabinoid/NAE tone in vivo. Furthermore, we examined nociception in these animals and demonstrate that the antinociceptive effects observed in these mice involve multiple receptors.

## Results

### Characterization of the endocannabinoid system in FABP5/7 KO mice

**Fig. 1:**
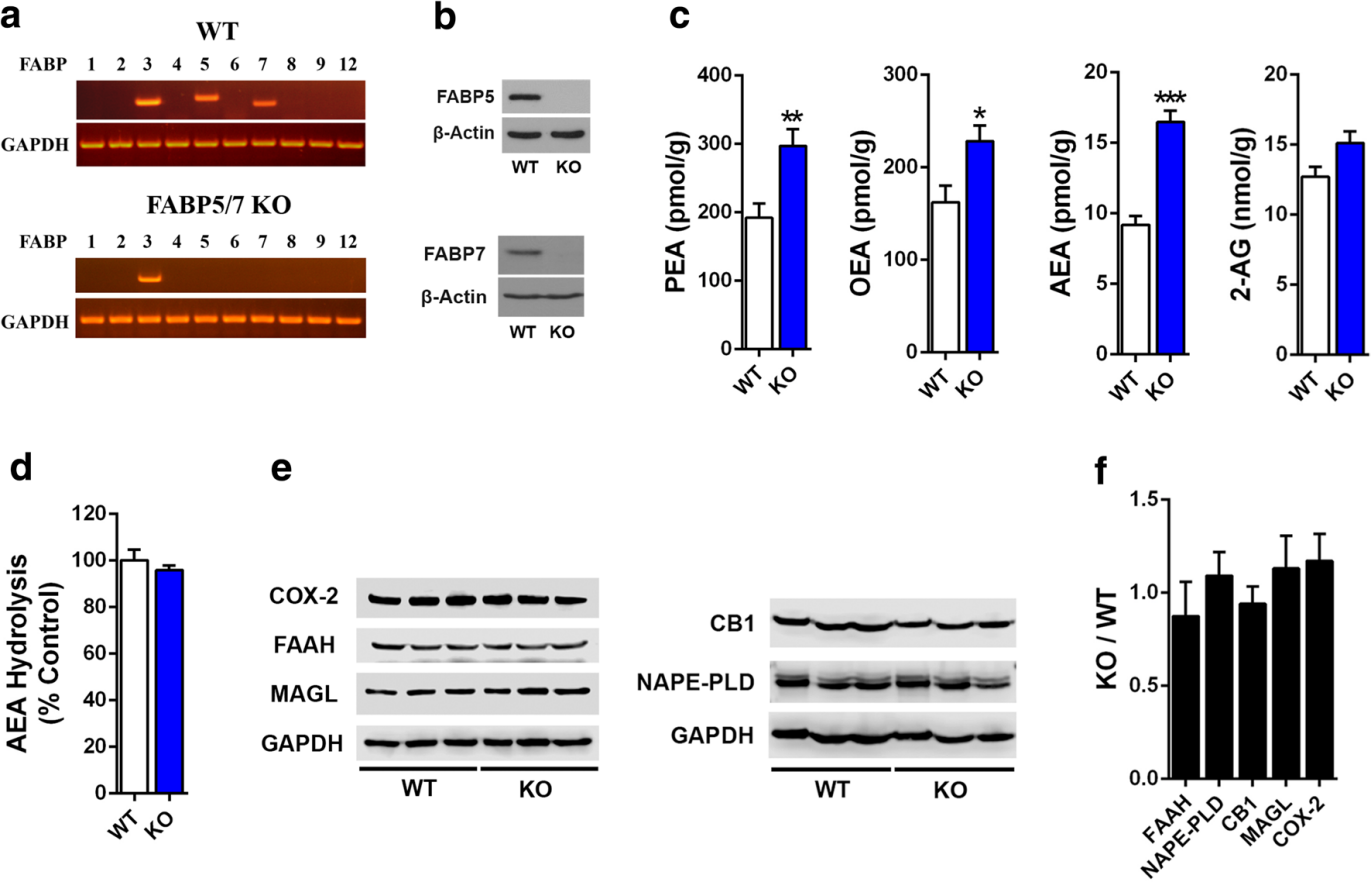
Characterization of the brain endocannabinoid system in FABP5/7 KO mice. **a** RT-PCR analysis of FABP expression in brains of WT and FABP5/7 KO mice. Note that of the ten FABP isoform profiled, selective deletion of FABP5 and FABP7 was observed in the brains of FABP5/7 KO mice. **b** Western blot confirms the absence of FABP5 and FABP7 in the brains of FABP5/7 KO mice. **c** Brain PEA, OEA, AEA, and 2-AG levels in WT and FABP5/7 KO mice. *p < 0.05; **p < 0.01; ***p < 0.001 (n = 6). **d** Hydrolysis of AEA in brain homogenates of WT and FABP5/7 KO mice. **e** Representative western blots of brain FAAH, MAGL, COX-2, NAPE-PLD, and CB1 expression in WT and FABP5/7 KO mice. **f** Quantification of western blots expressed as a ratio of fold change between FABP5/7 KO and WT mice

Generation of the FABP5/7 KO mice has been previously described [15]. We examined the expression of all ten FABP subtypes (FABP1-9 and FABP12) in the brains of WT and FABP5/7 KO mice. As expected, FABP3, FABP5, and FABP7 were expressed in WT brains and FABP5 and FABP7 were selectively deleted in FABP5/7 KO mice (Fig. 1a, b). Compensatory upregulation of other FABP subtypes was not observed in the brains of FABP5/7 KO mice (Fig. 1a). Previous work has shown that pharmacological FABP inhibition elevates brain endocannabinoid levels [6]. We confirmed that levels of the endocannabinoid AEA were likewise elevated in the brains of FABP5/7 KO mice (Fig. 1c). Furthermore, levels of the NAEs PEA and OEA were likewise elevated. 2-AG levels seemed slightly elevated but this did not reach statistical significance (p = 0.06). To confirm that the elevations in endocannabinoid/NAE levels were not due to changes in FAAH activity, AEA hydrolysis was examined in homogenates of brains from WT and FABP5/7 KO mice and no differences in AEA hydrolysis were observed between the genotypes (Fig. 1d). Lastly, we employed western blotting to examine changes in expression of proteins associated with the endocannabinoid system in FABP5/7 KO mice. There were no changes in CB1 receptor levels or in the expression of the endocannabinoid/NAE biosynthetic enzyme *N*-acyl phosphatidylethanolamine phospholipase D (NAPE-PLD) or the endocannabinoid catabolic enzymes FAAH, monoacylglycerol lipase (MAGL), and cyclooxygenase-2 (COX-2) (Fig. 1e, f). These data indicate that FABP5/7 KO mice possess elevated endocannabinoid/NAE levels, consistent with their role of gating the catabolism of endocannabinoids/NAEs by their respective enzyme(s).

### Nociception in FABP5/7 KO mice

**Fig. 2:**
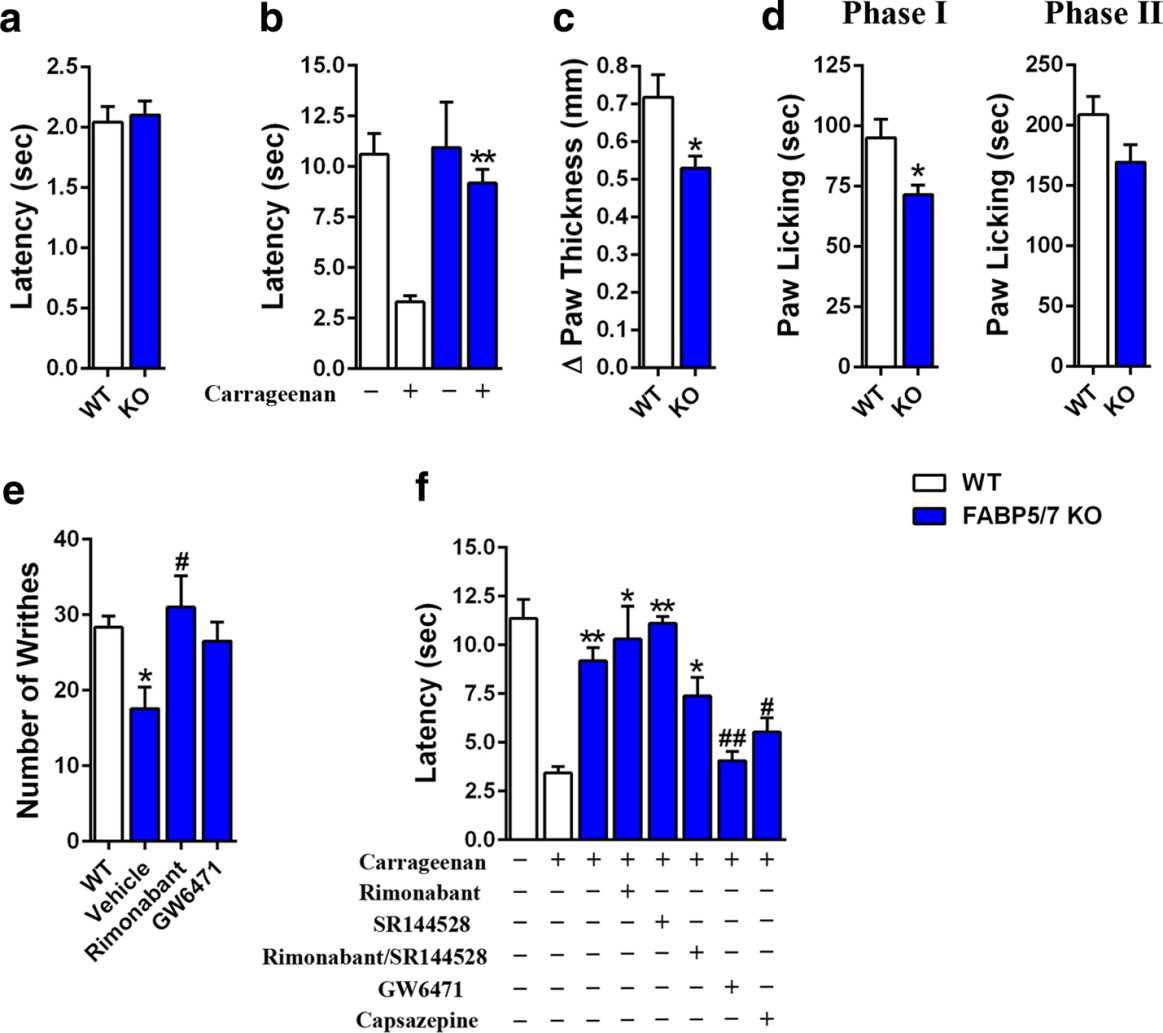
Nociception in FABP5/7 KO mice. **a** Tail withdrawal latencies of WT and FABP5/7 KO mice in the tail immersion test (n = 6). **b** Paw withdrawal latencies in the hargreaves test in WT (*white bars*) and FABP5/7 KO (*blue bars*) mice before and 4 h after carrageenan injection. **p < 0.01 versus carrageenan injected WT mice (n = 6). **c** Paw edema in WT and FABP5/7 KO mice after carrageenan injection (n = 6). **d** Nociception during the first (*left panel* 0–5 min) and second (*right panel* 15–60 min) phases of the formalin test. *p < 0.05 (n = 9). **e** Acetic acid writhing in WT and FABP5/7 KO mice. The FABP5/7 KO mice received a subcutaneous injection of vehicle, 3 mg/kg rimonabant, or 4 mg/kg GW6471 45 min before acetic acid injection. *p < 0.05 versus WT mice; ^#^p < 0.05 versus vehicle-treated FABP5/7 KO mice (n = 9–12). **f** Carrageenan-induced thermal hyperalgesia in WT and FABP5/7 KO mice treated with receptor antagonists. FABP5/7 KO mice were injected with vehicle, 3 mg/kg rimonabant, 3 mg/kg SR144528, 4 mg/kg GW6471, or 5 mg/kg capsazepine before carrageenan administration. *p < 0.05; **p < 0.01 versus carrageenan injected WT mice. ^#^p < 0.05; ^##^p < 0.01 versus carrageenan injected FABP5/7 KO mice (n = 9–12)

Baseline nociception was examined in WT and FABP5/7 KO mice. There were no differences in tail withdrawal latencies between the genotypes in the tail immersion test (Fig. 2a). Similarly, there were no differences in baseline thermal withdrawal latencies in the Hargreaves test (Fig. 2b). However, clear differences emerged in mice subjected to an inflammatory insult. In the carrageenan model of peripheral inflammation, WT mice developed thermal hyperalgesia and this was significantly attenuated in FABP5/7 KO mice (Fig. 2b). Furthermore, FABP5/7 KO exhibited reduced paw edema following carrageenan challenge (Fig. 2c).

We assessed FABP5/7 KO mice in two additional pain models. In the formalin test, FABP5/7 KO mice showed reduced nociception during the first phase of the test (Fig. 2d). In the acetic acid test, FABP5/7 KO mice showed an attenuated writhing response (Fig. 2e). The receptor(s) mediating the antinociceptive effects in FABP5/7 KO mice were subsequently examined. We focused upon CB1 and PPARα receptors because FABP5/7 KO mice possess elevated levels of NAEs that serve as ligands for both of these receptors. In the acetic acid writhing test, treatment of mice with the CB1 antagonist rimonabant or the PPARα antagonist GW6471 completely reversed the antinociceptive phenotype found in FABP5/7 KO mice (Fig. 2e). The involvement of these receptors was also examined in the carrageenan model. Consistent with the acetic acid test, GW6471 reversed the antinociceptive phenotype of FABP5/7 KO mice (Fig. 2f). Surprisingly, treatment of mice with rimonabant or the CB2 antagonist SR144528 alone or in combination did not block the analgesic effects observed in FABP5/7 KO mice.

Previous studies have demonstrated that activation of transient receptor potential vanilloid 1 (TRPV1) within the brain produces analgesia [16, 17]. Because AEA is a TRPV1 agonist [18] and its levels are elevated in FABP5/7 KO mice, we examined whether these receptors may likewise mediate the antinociceptive effects observed in FABP5/7 KO mice. Indeed, the TRPV1 antagonist capsazepine reduced thermal withdrawal latencies in FABP5/7 KO mice (Fig. 2f). These data indicate that FABP inhibition results in the upregulation of endocannabinoids/NAEs that produce analgesia by engaging multiple receptor systems.

**Fig. 3:**
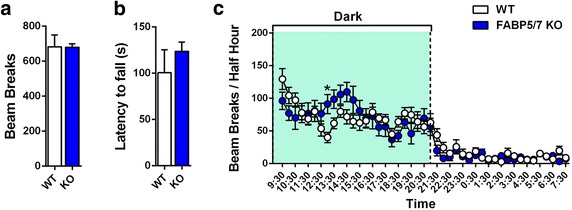
Motor activity in WT and FABP5/7 KO mice. **a** Locomotion in the open field test in WT and FABP5/7 KO mice (n = 6). **b** Latency to fall in the rotarod test for WT and FABP5/7 KO mice (n = 8–16). **c** Home cage activity over a 24-h period in WT and FABP5/7 KO mice. *p < 0.05 versus WT mice (n = 14)

The pain models employed herein measure evoked responses, effects that can be confounded by motor impairment. Consequently, we examined whether FABP5/7 KO mice exhibit motor deficits. In the open field test and rotarod tests, there were no differences between WT and FABP5/7 KO mice (Fig. 3a, b). We also profiled 24 h circadian home cage behavior and found no differences in home cage activity between the genotypes with the exception of enhanced locomotion in FABP5/7 KO mice at one time interval (Fig. 3c). These data indicate that FABP5/7 KO mice, similar to FABP inhibitor treated mice [6], do not possess motor deficits.

## Discussion

Endocannabinoids and NAEs reduce nociception through engagement of central and peripheral CB1 and PPARα receptors [11, 19]. Endocannabinoid inactivation proceeds through cellular uptake followed by intracellular hydrolysis [20]. Cytoplasmic FABPs transport endocannabinoids/NAEs to their catabolic enzyme(s) and are ideally situated to control endocannabinoid/NAE metabolism [4]. Consequently, therapeutic targeting of FABPs may serve as a novel strategy for the development of analgesic and anti-inflammatory drugs [6, 14].

Previous work by us and others has demonstrated that inhibition of FABPs reduces the cellular uptake of endocannabinoids [4, 21]. Consequently, pharmacological FABP inhibition and genetic FABP5 ablation results in elevated AEA levels [6, 13]. Herein we confirm that mice lacking FABP5 and FABP7, the brain-expressed FABPs with highest affinities for endocannabinoids/NAEs show markedly elevated AEA levels. The relatively similar elevations in AEA levels between FABP5/7 KO mice and FABP5 KO mice [13] suggests that the contribution of FABP7 towards regulating the AEA tone may be minimal. This is consistent with the low expression level of FABP7 in the adult mouse brain [22]. In addition to AEA, we demonstrate for the first time that ablation of FABPs results in elevated PEA and OEA levels, indicating that FABPs are important regulators of the NAE tone in vivo.

We have recently reported that mice treated with FABP5 and FABP7 selective inhibitors display antinociceptive effects when subjected to diverse pain models [6, 14]. In support of this, we demonstrate here that mice lacking FABP5 and FABP7 possess a similar antinociceptive phenotype. Similar to acute pharmacological FABP inhibition, the antinociceptive effects in FABP5/7 KO mice are mediated by CB1 and PPARα receptors. Unexpectedly, blockade of CB1 receptors with rimonabant failed to reverse the analgesic effects in the carrageenan model, which contrasts to our previous results following acute pharmacological FABP inhibition [14]. This may reflect possible adaptive changes in response to chronic endocannabinoid elevation, although the exact mechanism responsible for this discrepancy requires further elucidation. Although rimonabant also engages TRPV1 [23], the use of identical rimonabant doses in our prior and current study suggest that its lack of efficacy is unlikely to stem from off-target effects at TRPV1. Furthermore, using a TRPV1 antagonist, we provide evidence that TRPV1 receptors likewise mediate the analgesic effects observed in FABP5/7 KO mice, consistent with an established role of supraspinal TRPV1 in pain modulation [16, 17]. Although we hypothesize that this effect may be mediated by elevated levels of the TRPV1 agonist AEA, it is likewise possible that other FABP-regulated TRPV1 ligands may mediate this effect.

## Conclusions

In summary, this study provides evidence that FABP5 and FABP7 contribute to endocannabinoid/NAE metabolism in vivo and establishes these proteins as important regulators of the NAE and endocannabinoid tone. The observation that FABP5/7 KO mice display inflammation-associated analgesia but unchanged baseline thermal withdrawal latencies suggests that inflammation unmasks a role for FABP-regulated ligands in pain modulation. Therefore, pharmacological agents that selectively disrupt FABP function may serve as novel analgesics. Future studies aimed at characterizing the contribution of peripherally and centrally expressed FABPs toward nociception are required to conclusively delineate the anatomical site(s) of FABP-mediated analgesia.

## Methods

### Chemicals and drugs

PEA, *d*_*4*_-PEA, OEA, *d*_*2*_-OEA, 2-AG and *d*_*5*_-2-AG were from Cayman Chemical while AEA and *d*_*4*_-AEA were from R&D systems. [^14^C]AEA, rimonabant and SR144528 were obtained from the Drug Supply Program at the National Institute on Drug Abuse. GW6471 was purchased from Sigma while capsazepine was purchased from Cayman Chemical.

### Animals

Male C57Bl/6 mice (2–3 months old) were purchased from Jackson Laboratories. FABP5/7 KO mice were previously described [15]. The animals were group housed and had ad libitum access to food and water. The animals were habituated to handling in the experimental room for at least 1 day before each experimental session. The experiments were approved by the Stony Brook University Institutional Animal Care and Use Committee (#277150).

### Drug administration

Receptor antagonists were injected 30 min before behavioral measurements and were administered in a volume of 10 µl/g body weight. Rimonabant and SR144528 (3 mg/kg) were dissolved in saline containing 5 % ethanol and 5 % cremophor-EL. GW6471 (4 mg/kg) was dissolved in saline containing 2 % DMSO and 5 % cremophor-EL. Capsazepine (5 mg/kg) was dissolved in saline containing 2 % DMSO and 10 % Tween 80. The antagonists were administered via the intraperitoneal route with the exception of the acetic acid test where the drugs were injected subcutaneously.

### Nociceptive tests

The formalin, acetic acid writhing, and carrageenan-induced inflammatory pain models were performed exactly as described [6]. For the tail immersion test, mice were gently restrained and one cm of the tail was submerged in a water bath set at 56 °C. The latency to withdraw the tail from the water bath was measured using a stopwatch.

### Motor tests

In the open field test, mice were placed in the open field chamber containing a laser beam grid (San Diego Instruments) and beam breaks indicative of locomotion were scored over a 5 min period. The rotarod test using a rotarod apparatus (Rotamex 0192-100 M, Columbus Instruments) was used to examine motor coordination by measuring the ability of mice to remain on a rotating rod. During each trial, the rotating rod accelerated a linear rate from a set starting rate of 4 RPM to a set ending rate of 40 RPM, all within a 5 min time frame. Subjects were observed throughout the test, and falls from the axle, including time and fall speed, are automatically recorded by the apparatus. Subjects that cling to the axle—remaining stationary as they rotate with the axle—are recorded as a “passive rotation”. Latency to fall or commit a passive rotation is measured by the apparatus. Subjects whose latency to fall or commit a passive rotation exceeds the designated 5 min “passes” the given trial. Subjects who fail to do so “fail” the trial. Home cage circadian activity was measured for each mouse for a period of 72 h. The first 24 h acted as a habituation period and no measurements were taken. Each half-hour time point from the two recorded days was averaged together for each subject.

### Lipid quantification

Endocannabinoid/NAE quantification was performed exactly as described [6].

### Enzyme assays

AEA and 2-OG hydrolysis was performed exactly as described [6].

### RT-PCR

Mouse brains were homogenized and cDNA synthesis commenced as previously described [5]. The cDNA was amplified using LongAmp Taq DNA polymerase (New England Biolabs) using gene specific primers. The following primers were used: FABP1: 5′-TCATGAAGGCAATAGGTCTG-3′ and 5′-GTTCAGTCACGGACTTTATGC -3′; FABP2: 5′-GAAAATGGGCATTAATGTGATGA-3′ and 5′-AGAAACCTCTCGGACAGCAA-3′; FABP3: 5′-CATCGAGAAGAACGGGGATA-3′ and 5′-TGCCATGAGTGAGAGTCAGG-3′; FABP4: 5′-AGTGGCAGGCATGGCCAAGC-3′ and 5′-GTCACCATCTCGTTTTCTC-3′; FABP5: 5′-AGGAAGATGGCTGCCATGG-3′ and 5′-TGTTCATGACACACTCCAC-3′; FABP6: 5′-CATGAAGCGCCTGGGTCT-3′ and 5′-AACTTGTCACCCACGACCTC-3′; FABP7: 5′-AGTGGGAAACGTGACCAAAC-3′ and 5′-TTTCTTTGCCATCCCACTTC-3′; FABP8: 5′-CTACATGAAAGCTCTAGGTGTGG-3′ and 5′-TCTCCAGTGTCACGATGCTC-3′; FABP9: 5′-GTGAGAGAACTGGGAGTGGAAT-3′ and 5′-AGCCATTTTTGGACCTGGAT-3′; FABP12: 5′-ATGAAGGAATTGGGAGTAGGAAG-3′ and 5′-CCTGGACTTGAACCAAGGAG-3′; GAPDH: 5′-CGAGACCCCACTAACATCAAA-3′ and 5′-CTTCCACAATGCCAAAGTTGT-3′.

### Western blotting

Western blot experiments were performed as described [5]. Blots were probed with the following antibodies: COX2 (1:1000, Abcam #Ab15191), FAAH (1:1000, Abcam #Ab54615), MAGL (1:400, Cayman Chemical #10212), GAPDH (1:5000, Abcam #Ab8245), CB1 (1:1000, Abcam #Ab172970), NAPE-PLD (1:400, Abcam #Ab95397), FABP5 (1:1000, BioVendor R&D #RD181060100), or FABP7 (1:200, Abcam #Ab32423). The blots were developed using the Immun-star HRP substrate (Bio-Rad) and scanned using a C-DiGiT scanner (Li-COR). Protein band intensities were quantified and normalized to the respective GAPDH intensities. To quantify changes in protein expression between WT and FABP5/7 KO mice, ratios of normalized protein intensities were compared between the genotypes.

### Statistical analysis

Behavioral data are presented as mean ± SEM of at least six animals per group. Biochemical data are presented as mean ± SEM of at least three independent experiments performed in triplicate. Statistical significance was determined using unpaired t-tests or one-way ANOVA followed by Dunnett or Tukey post hoc analyses as appropriate. Home cage activity was analyzed by two-way ANOVA followed by Bonferroni post hoc test. In all cases, differences of p < 0.05 were considered significant.

## Abbreviations

FABP: fatty acid binding protein
FAAH: fatty acid amide hydrolase
CB1: cannabinoid receptor 1
CB2: cannabinoid receptor 2
PPARα: peroxisome proliferator-activated receptor alpha
MAGL: monoacylglycerol lipase
NAPE-PLD: *N*-acyl phosphatidylethanolamine phospholipase D
COX2: cyclooxygenase-2
RT-PCR: reverse transcription polymerase chain reaction
